# Metatranscriptomic Analysis of the Bacterial Symbiont *Dactylopiibacterium carminicum* from the Carmine Cochineal *Dactylopius coccus* (Hemiptera: Coccoidea: Dactylopiidae)

**DOI:** 10.3390/life9010004

**Published:** 2019-01-03

**Authors:** Rafael Bustamante-Brito, Arturo Vera-Ponce de León, Mónica Rosenblueth, Julio César Martínez-Romero, Esperanza Martínez-Romero

**Affiliations:** 1 Center for Genomic Sciences, Universidad Nacional Autónoma de México, UNAM, Cuernavaca, Morelos C.P. 62210, Mexico; rbustab@ibt.unam.mx (R.B.-B.); veraponcedeleon.1@osu.edu (A.V.-P.d.L.); mrosen@ccg.unam.mx (M.R.); jmartine@ccg.unam.mx (J.C.M.-R.); 2 Department of Ecology, Evolution and Organismal Biology, The Ohio State University, Columbus, OH 43210, USA

**Keywords:** scale insect, *Opuntia*, endosymbiont, betaproteobacteria, nitrogen-fixation: gut microbiota, polysaccharide catabolism

## Abstract

The scale insect *Dactylopius coccus* produces high amounts of carminic acid, which has historically been used as a pigment by pre-Hispanic American cultures. Nowadays carmine is found in food, cosmetics, and textiles. Metagenomic approaches revealed that *Dactylopius* spp. cochineals contain two *Wolbachia* strains, a betaproteobacterium named *Candidatus* Dactylopiibacterium carminicum and *Spiroplasma*, in addition to different fungi. We describe here a transcriptomic analysis indicating that *Dactylopiibacterium* is metabolically active inside the insect host, and estimate that there are over twice as many *Dactylopiibacterium* cells in the hemolymph than in the gut, with even fewer in the ovary. Albeit scarce, the transcripts in the ovaries support the presence of *Dactylopiibacterium* in this tissue and a vertical mode of transmission. In the cochineal, *Dactylopiibacterium* may catabolize plant polysaccharides, and be active in carbon and nitrogen provisioning through its degradative activity and by fixing nitrogen. In most insects, nitrogen-fixing bacteria are found in the gut, but in this study they are shown to occur in the hemolymph, probably delivering essential amino acids and riboflavin to the host from nitrogen substrates derived from nitrogen fixation.

## 1. Introduction

Scale insects may have evolved in the Jurassic and Triassic periods [1], hence around 150–250 million years ago. Most scale insect species feed on plant sap, and many, though not all, are considered plant pests. Similar to other invertebrates, scale insects may contain specialized cells called bacteriocytes, which harbor bacterial symbionts inherited by their progeny [2]. Endosymbionts are largely dependent on their hosts, have reduced genomes, and provide amino acids or vitamins to the insects. In scale insects, flavobacterial endosymbionts are commonly found with other co-symbionts that in most cases are gammaproteobacteria, and in few cases *Wolbachia* (reviewed in [2]).

The carmine cochineal (*Dactylopius coccus*) is a scale insect that feeds on the sap of *Opuntia* cactus and several other cacti. It has commercial value [3], as it is used to produce the carmine dye for food, textiles, cosmetics, and pharmaceutics, as well as for art and crafts. In the insect, carminic acid may function as a defense substance against predators [4,5]. This red dye has been used by pre-Hispanic cultures for a long time, since *D. coccus* was domesticated at least 1000 years ago [6], and it is still controversial whether domestication occurred in Peru or Mexico [7,8]. Carmine has remained in use as it is considered innocuous, while other dyes have adverse health effects [6]. Other wild *Dactylopius* species also produce carminic acid, but in reduced quantities and quality, and the insects are more aggressive plant pests [6,9]. The cochineal feeds on the cactus phloem that contains sugars and some amino acids [10], as well as soluble polysaccharides, proteins, peptides, and even RNAs [11,12,13,14]. Like many scale insects, cochineals exhibit sexual dimorphism [1]. Females have four developmental stages: eggs, first instar nymph, second instar nymph, and adults that are sessile. Adult females have a length of around 4–6 mm. Males have six developmental stages. Under optimal environmental conditions, the female cycle lasts around three months [15].

Notably, the cochineals *Dactylopius* spp. do not contain flavobacterial endosymbionts or bacteriomes (special organs with insect cells that contain bacterial endosymbionts), as other scale insects do; they instead harbor a betaproteobacterium named *Candidatus* Dactylopiibacterium carminicum (hereafter abbreviated as *Dactylopiibacterium*), two strains of *Wolbachia* (*w*DacA and *w*DacB), and a *Spiroplasma* [16,17], in addition to various fungi [18]. Other bacteria that have been sporadically found may represent transient gut bacteria that were acquired from the host plant [19].

*Dactylopiibacterium* was detected in all tested individuals from the domesticated *D. coccus*, from the wild species *Dactylopius ceylonicus*, *D. confusus*, *D. opuntiae*, and *D. tomentosus* [17], and in the ovaries of *D. coccus* and *D. opuntiae* [19]. The genomes of *Dactylopiibacterium* recovered from the metagenomes from females and males of *D. coccus*, as well as those recovered from the wild species *D. opuntiae* were published [17]. Due to their large genome, *Dactylopiibacterium* seems to be a recently acquired cochineal symbiont [17]. *Dactylopiibacterium* has not been isolated in culture media, and is phylogenetically placed in the betaproteobacteria family of Rhodocyclales, closely related to *Azoarcus*, an efficient nitrogen-fixing endophyte of rice and grasses [20]. Nitrogen fixation and expression of the nitrogenase subunit *nifH* gene (evaluated by reverse transcription-polymerase chain reaction, RT-PCR) were detected in the hemolymph and ovaries from *Dactylopius* spp. [17]. In insects such as ants, termites, bark beetles, and fruit flies that feed on high carbon and low nitrogen diets, there are also nitrogen-fixing bacteria that are located in the gut [21,22,23,24,25,26]. *Dactylopiibacterium* may also synthesize amino acids and vitamins. However, little is known of the function of the symbionts from the carmine cochineal. We report here a metatranscriptomic study from dissected gut and ovaries, as well as from the hemolymph of the cochineal *D. coccus*, shedding light on the metabolic functions of *Dactylopiibacterium* in its host.

## 2. Materials and Methods

### 2.1. Tissue Dissection, RNA Extraction, and Sequencing

**Figure 1:**
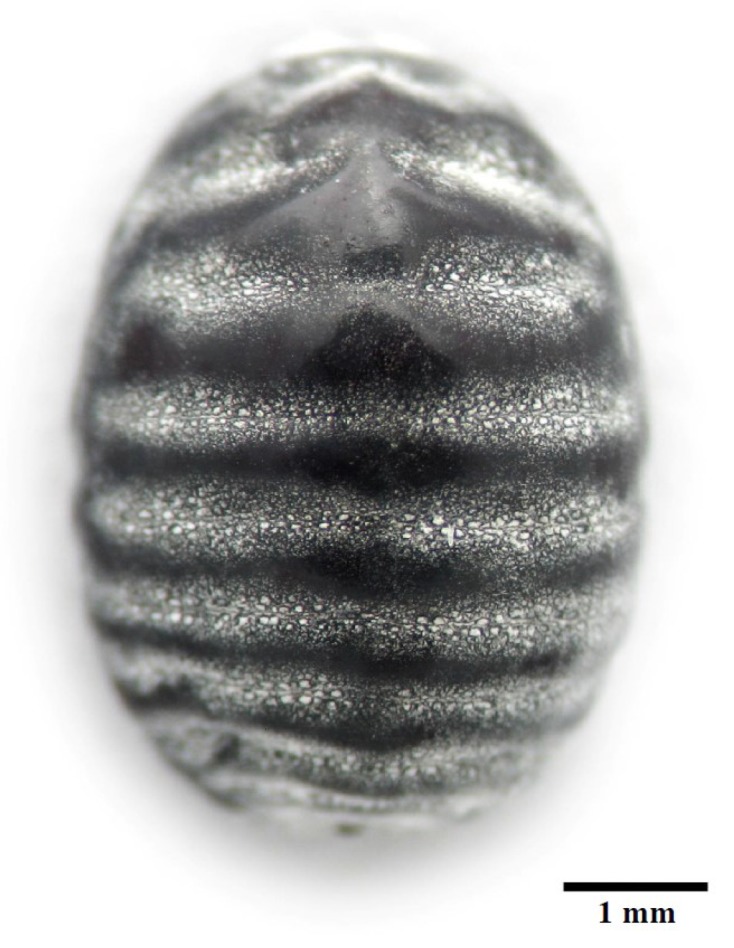
Dorsal view of female second instar nymph of *Dactylopius coccus* with wax removed with 96% ethanol.

Second instar nymphs of female cochineals (Figure 1) were detached from cactus and rinsed with ethanol 90%. Here we used a pool of 30 s instar nymphs for each replicate, and three replicates were used. However, each individual may have been in a slightly different stage, because they were not synchronized populations. The cochineals were dissected using autoclaved forceps cleaned with RNaseZAP (Qiagen, Hilden, Germany) to remove whole gut (including foregut, midgut, hindgut, and Malpighian tubules) and ovaries, and hemolymph was collected (three samples from the same individuals) with RNAse-free pipette tips. Before dissecting the gut and ovaries, insects were bled from the dorsal thorax with a sterile syringe to collect the hemolymph. The dissected guts and ovaries were rinsed with sterile phosphate-buffered saline, (PBS) to remove remnant hemolymph. Tissues were maintained on ice with RNAlater, an RNA stabilizing reagent (Qiagen) that was subsequently removed.

A modified RNEeasy (Qiagen) procedure with lysozyme and DNAse I was used as reported by Guerrero-Castro et al. [27]. RNA was quantified by Nanodrop and visualized in 1% electrophoresis gels. RNA integrity was analysed with an Agilent 2200 TapeStation (Santa Clara, CA USA). High-quality RNA samples were used for strand-specific library preparation by TruSeq Stranded Total RNA kit (Illumina), and rRNA was removed with the RiboZero Removal kit for Bacteria (Illumina). Nine libraries were generated, three for each tissue used (hemolymph, gut, and ovary), and they were sequenced using one lane on an Illumina HiSeq4000 sequencer (Macrogen, Korea) with a 100 bp read length pair end protocol.

### 2.2. Bioinformatic Analyses

#### 2.2.1. Mapping to Reference Genome, Core Transcriptome, and Differential Expression Analysis

The quality of raw reads was inspected with FASTQC v0.11.6 (https://www.bioinformatics.babraham.ac.uk/projects/fastqc/). We found that all reads had a quality above 32 Phred quality score. Even though all libraries were subjected to ribosomal depletion, after quality-check, small (SSU) and large (LSU) subunits of ribosomal gene sequences were detected in the samples, and manually removed by home-made Perl and bash scripts. All high-quality reads were mapped to *Dactylopiibacterium* NFE1 genome (MQNN00000000) using bowtie2 v2.3.0 [28] (parameters: --very_sensitive -1 x_R1.fq -2 x_R2.fq -p 30). Mapped reads from each sample were independently recovered and transformed to BAM files by samtools v1.7 parameters: view –bS. Relative abundance (in percentage of mapped reads) of *Dactylopiibacterium* transcripts present in each tissue were calculated from BAM stats files obtained by the samtools stats command. Subsequently, sorting for position was done with parameter sort to obtain the files x.bam.sorted [29]. Artemis was used to obtain the total raw transcripts counts from the mapped reads against NFE1 genome [30] using the BAM files as well as the fasta and gene feature format (gff) files. BAM files were deposited as an NCBI bioproject under accession number PRJNA355137.

For gene expression level quantification, transcripts per million (TPM) values were calculated with RSEM v1.2.31 pipeline [31], using the pair-mapped transcripts from BAM files described above and a gene-transfer format (GTF) file from the NFE1 *Dactylopiibacterium* genome. Total TPM abundance–expression matrices for each replicate of all treatments were obtained by the abundance_estimates_to_matrix.pl Trinity v2.5.1 utility script, using the RSEM gene-result files. Average log2TPM were manually obtained from TPM-abundance matrices and merged in a single average-log2TPM matrix.

Differentially expressed genes between gut and hemolymph samples were obtained with a NOISeq v2.24.0 Bioconductor package. Raw counts of mapped transcripts data were normalized using the trimmed mean of M-values method (TMM). Differentially expressed genes were selected with the following criteria: false discovery rate (FDR) threshold of 0.95, adjusted *p*-values equal or below 0.05, and absolute fold changes over 1 [32]. Due to the low number of transcripts, ovary samples were not included in any of the differential expression analyses, though we determined which genes were expressed in this tissue and included them in the flagella gene expression analysis.

#### 2.2.2. Metabolic Analysis and Construction of a Metabolic Model

To construct a *Dactylopiibacterium* metabolic and membrane protein model from expression genes, mapped reads were assigned to genes, and those genes that were expressed in two of the three replicates from each tissue were retained. All genes were sorted and grouped by clusters of orthologous group (COG) categories. For this, all positive expressed genes were blasted to COG database (https://www.ncbi.nlm.nih.gov/COG/). Once classified, iPATH3 (Interactive Pathways Explorer v3, https://pathways.embl.de/) was used to construct the metabolic maps from each tissue [33]; these were used to construct a *Dactylopiibacterium* core metabolism using predesigned images [34]. Additionally, for enzymatic and metabolic pathway categorizing, all expressed genes were annotated with the Kyoto Encyclopedia of Genes and Genomes (KEGG) database using BlastKoala [35]. Enzymatic and protein annotation from each gene were retrieved from their corresponding KO numbers using the KEGG REST server package from Bioconductor (https://bioconductor.org/packages/release/bioc/html/KEGGREST.html). The presence or absence of genes for particular metabolic pathways (i.e., flagella production or secretion system) from different *Dactylopius* tissues were plotted using the KEGG Mapper-Reconstruct pathway tool (https://www.kegg.jp/kegg/tool/map_pathway.html).

#### 2.2.3. Quantitative Analysis

**Figure 2:**
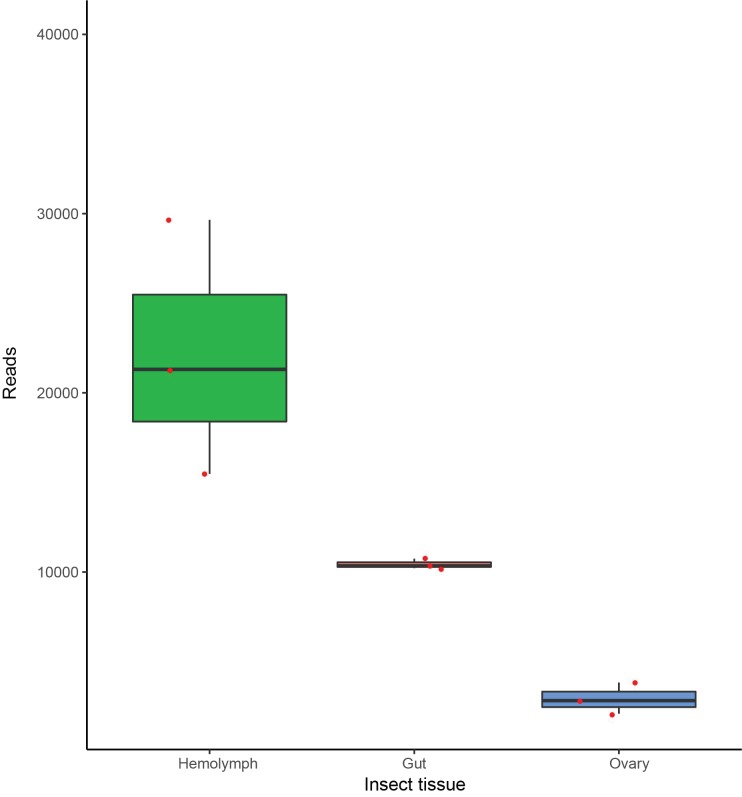
Number of *Dactylopiibacterium* expressed transcripts in different *Dactylopius* tissues. Reads were mapped to *Dactylopiibacterium* NFE1 reference genome.

*Dactylopiibacterium* transcript-reads (selected as described above in Section 2.2.1) were used to calculate the ratios of the sum of read numbers obtained in total from the hemolymph and the gut shown in Figure 2. Ratios were obtained as well by comparing each gene in pairs of distinct tissues (gut/hemolymph, hemolymph/gut) by dividing the sum of reads from hemolymph or gut by the sum of reads from gut or hemolymph, respectively. In cases where the divisor was 0, it was changed to one, since division by zero is not defined. The number of reads from the hemolymph were adjusted (see Results). Ratios were also numerically ordered and ranked. 

## 3. Results

### 3.1. Dactylopiibacterium Expression in the Carmine Cochineal with Core Transcriptome Analysis

**Figure 3:**
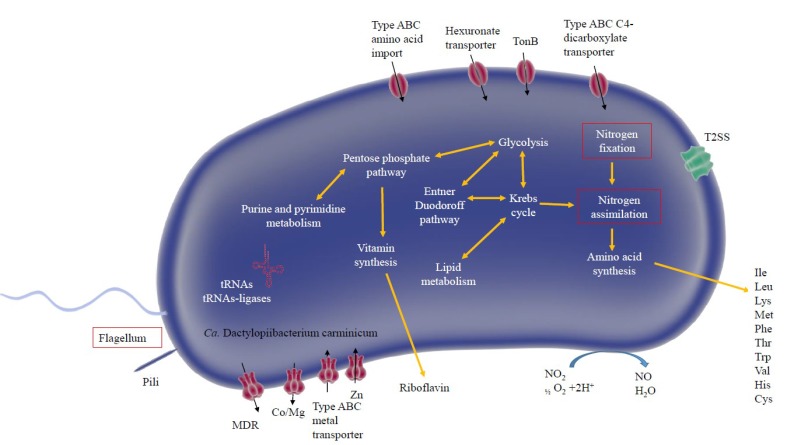
Predicted metabolism and cellular features of *Dactylopiibacterium* in the insect host (*Dactylopius*). Red boxes represent expressed metabolism genes in the gut and hemolymph.

A low proportion of transcripts in metatranscriptomes from the hemolymph, gut, and ovary were from *Dactylopiibacterium* (0.07%, 0.04%, and 0.01%, respectively)—most transcripts were from the cochineal insect and from *Wolbachia*. In total, the numbers of *Dactylopiibacterium* transcripts recovered from hemolymph, gut, and ovary were 66442, 31312 and 8759, respectively (Figure 2), thus we estimated that there were 2.1 times more reads from hemolymph than from gut and 7.5 times more reads from hemolymph than from ovary. 2083, 1867, and 972 genes expressed in insect hemolymph, gut, and ovary, respectively, were used to reconstruct the biochemical pathways, whereas genes expressed in common were used to depict a core of *Dactylopiibacterium* functions in the cochineal, and are marked in red (Figure 3).

Transcripts found in the gut and hemolymph were from genes encoding enzymes of the glycolytic pathway, Krebs cycle, all amino acids (except asparagine), purine and pyrimidine biosynthesis, all ribosomal proteins, and aminoacyl tRNA ligases. Other common transcripts were from genes encoding chaperonin GroEL, elongation factor Tu, FtsH, and FtsZ for bacterial cell division. Additional transcripts were for enzymes involved in galacturonic acid metabolism, namely D-altronate hydrolase, tagaturonate reductase and glucuronate isomerase (UaxA (EC 4.2.1.8), UaxB (EC:1.1.1.58) and UaxC (EC:5.3.1.12), respectively; Table S1a), constituting a biochemical pathway that ends in the production of glyceraldehyde 3-phosphate and consequently leads to pyruvate. In the hemolymph and gut, expressed genes encode transcriptional regulators and genes encoding rhamnogalacturonan lyases (EC:4.2.2.2; Table S1a).

Nitrogen-fixing capability has been previously associated with *Dactylopiibacterium* [17]. COG category analysis showed the presence of signal transduction histidine kinase (NtrY) involved in nitrogen fixation and metabolism regulation (COG 5000) in all the tissues. Additionally, an expression level of 2.17 and 5.17 log2TPM for the gene encoding the catalytic nitrogenase molybdenum iron protein (*nifD*) was observed from hemolymph and ovary respectively (Table S1a). The analysis of nitrogen-fixing associated genes showed the presence of the nitrogenase electron donor ferredoxin *fixA* and Pi II nitrogen sensor protein also in hemolymph (Table S1a). Previously *nifH* gene expression was detected in hemolymph and ovary [17]. Furthermore, genes for molybdenum and iron transporters (ABC-transporter and permeases) were expressed in the gut and hemolymph (Table S1a). In the ovary, expression of iron transporters was detected (Table S1a).

**Figure 4:**
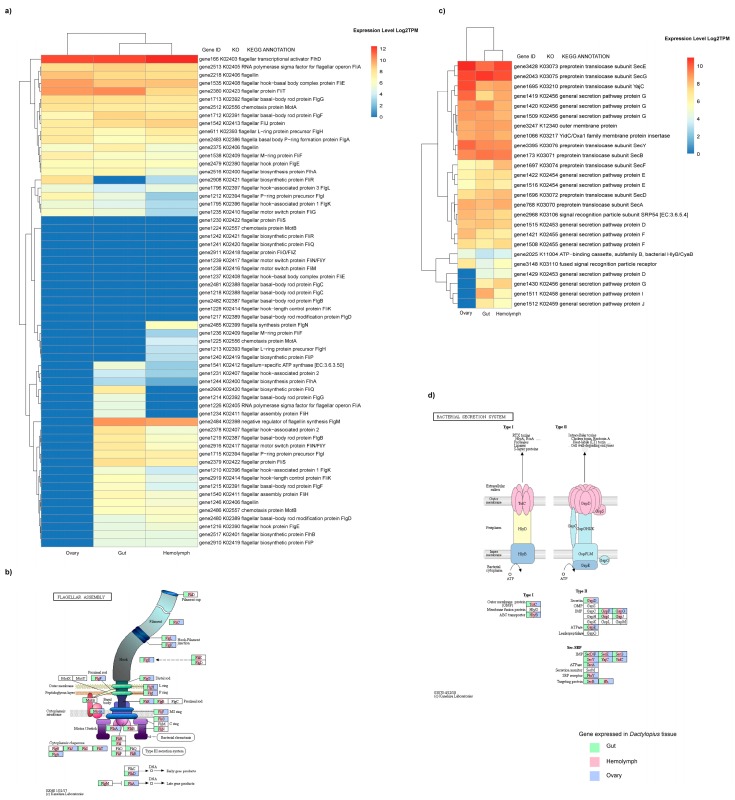
Flagellar and secretion systems expressed by *Dactylopiibacterium* in their insect host. (**a**) Heat map showing the average expression level of *Dactylopiibacterium* genes for flagellar production and motility in *Dactylopius*. (**b**) Flagellar diagram showing the expression (highlighted) of different flagellar components by *Dactylopiibacterium* in different insect tissues. (**c**) Heat map showing the average expression level of *Dactylopiibacterium* genes for secretion systems 1 and 2 in *Dactylopius.* (**d**) Bacterial secretion system diagram showing the expression (highlighted) of different *Dactylopiibacterium* genes for SS1 and SS2 in different insect tissues.

Expression analysis revealed high TPM values of genes for the *Dactylopiibacterium* proteins involved in flagellar assembly (Figure 4a,b). In particular, the transcriptional regulator for flagella (*flhD*) is highly expressed in all three tissues sampled (Figure 4a,b, Table S1a). However, the flagellar biosynthesis regulator anti-σ-factor *flgM* is only expressed in the gut and hemolymph (Figure 4a,b, Table S1a). Flagellar motor proteins also showed different expression patterns; in hemolymph the three canonical genes for flagellar rotation and motor switching (*fliG*, *fliM* and *fliN*) were highly expressed (log2TPM > 3, Figure 4a,b; Table S1a). In the gut *fliG* and *fliN* were also expressed, and in the ovary only *fliN* transcripts were observed. This evidence suggests that bacteriaare motile in hemolymph. Other genes related to motility and intracellular trafficking, secretion, and vesicular transport (COG N and U categories) were found expressed in all tissues. Multiple genes related to Type IV pili (Tfp) were found expressed in all tissues (Figure S1a), while genes *pilO*, *pilN*, and *pilP* were expressed only in the ovary. The chemotaxis *che*A gene was found expressed in all three *Dactylopius* tissues sampled. The *cheBD* and *cheVYW* genes were transcribed in the gut and hemolymph, respectively.

Genes for two different secretion systems were also expressed in *Dactylopiibacterium*. Particularly, alpha-hemolysin/cyclolysin transport system (hlyB, cyaB) and the outer membrane protein (TolC) of the bacterial secretion system 1 were highly expressed in *Dactylopius* hemolymph, gut, and ovary (Figure 4c,d; Table S1a). Additionally, genes for the ATPase (GspE) and general secretion pathway proteins F, G, and D (GspF, GspG, and GspD) associated with the bacterial secretion system 2 were expressed in all tissues (Figure 4c,d; Table S1a). Genes for general secretion pathway proteins I and J (GspI and GspJ) were also found in the hemolymph and gut (Figure 4c,d; Table S1a).

**Figure 5:**
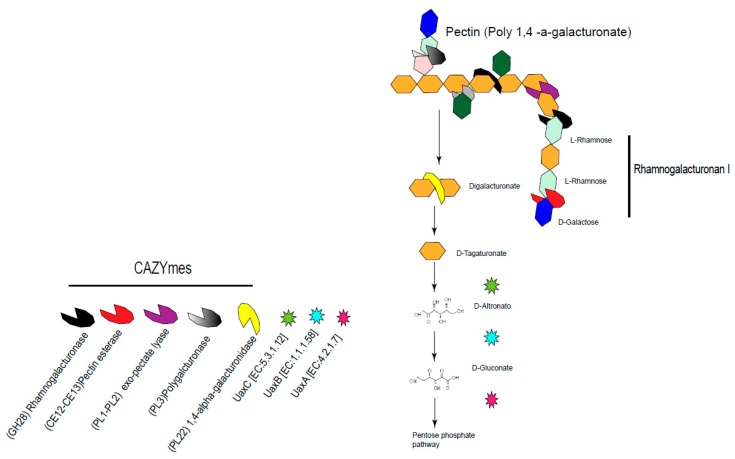
Expressed CAZymes and metabolic genes involved in pectin and rhamnogalacturonan metabolism of *Dactylopiibacterium* in the insect gut.

The carbohydrate-active enzyme (CAZyme) analysis [36] was used to define polysaccharide catabolic domains. The CAZyme toolkit revealed domains for exo-pectate lyases (PL; EC 4.2.2-) PL1 and PL3, as well as an exo-polygalacturonase (PL2) and for rhamnogalacturonan endolyase (PL11) and oligogalacturonate lyase (PL22). Additionally, some glycosyl hydrolases (GH; EC 3.2.1), and endoglucanase (GH74) were found expressed (Table S1b, Figure 5). This suggests that *Dactylopiibacterium* can actively metabolize plant polysaccharides, such as pectin and rhamnogalacturonan I. Furthermore, glycerol may be an additional carbon source for *Dactylopiibacterium*.

### 3.2. Differential Expression Analysis with NOISeq

With NOISeq, a total of 207 differentially expressed genes were obtained between the gut and hemolymph samples—116 up-regulated in the gut and 91 in the hemolymph (Table S2). Among the up-regulated genes in the gut samples, the pilus assembly protein Tfp was found (suggesting *Dactylopiibacterium* uses pili in this tissue), as well as the LysR transcriptional regulator and some transporter genes involved in drug resistance (MdtA). Whereas in the hemolymph samples, highly expressed genes were involved in carbohydrate metabolism (glycolysis and pentose pathway) and cellular replication (segregation/condensation protein A, septum formation inhibitor Maf) (Table 1).

### 3.3. Dactylopiibacterium Quantitative Transcript Analysis

**Figure 6:**
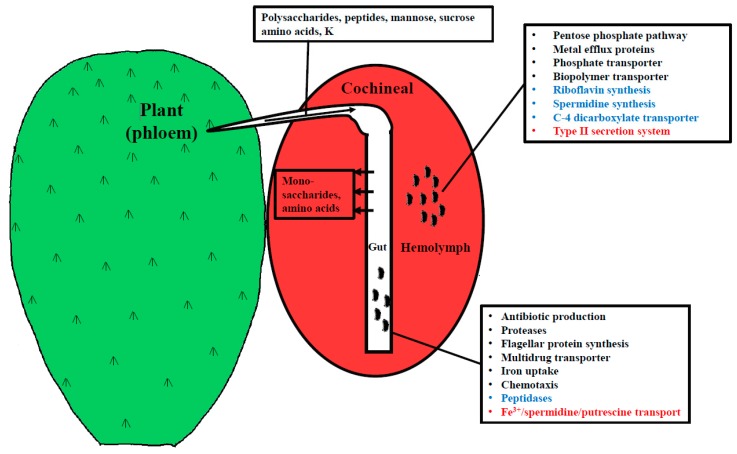
Illustrative proposed functional markers depicting *Dactylopiibacterium* differentially expression in different tissues: in blue, from the quantitative analysis; in red, from NOISeq; and in black, from both analyses.

To expand the results obtained so far, an additional approach was implemented. As there were more *Dactylopiibacterium* reads from hemolymph than from gut or ovary (Figure 2), it was expected that in hemolymph there would be more ribosomal-protein transcripts, and indeed these were 2.9 times more abundant in comparison to gut. Thus, we used this value to equiparate the number of reads from hemolymph to those from gut, by dividing hemolymph reads by 2.9. With this adjustment, all genes that were equally expressed attained ratios around one as observed for genes encoding ribosomal-proteins (except gene 3388, Table S3a), aminoacyl-tRNA ligases (with the exception of that for glutamate), elongation factor Tu, ATP synthase subunits and cell division proteins FtsH and FtsZ (not shown). Arbitrarily, we defined here that up-regulated genes had ratios of 2 or above 2 and listed them in Table S3b,c. Functional markers were proposed for gut and hemolymph based on this quantitative analysis and NOISeq (Figure 6). When comparing to ovaries, the ratio of hemolymph reads was around seven using ribosomal protein or aminoacyl-tRNA ligase transcript-numbers. 

Among all genes, we particularly note transcriptional regulators that were differentially expressed in gut and hemolymph (Table S3b,c), that would lead to further differences in gene regulation. In the gut, these encode ArsR (for metal tolerance, see below), BolA (a master regulator involved in biofilm formation [37]), Com, and YebC, which were not found expressed in the hemolymph. In hemolymph, transcription regulator genes that were found to be highly expressed encode AraC, AsnC, GntR, and NdrR.

## 4. Discussion

It is a common observation that some bioinformatic programs for differential gene analyses in transcriptomics yield varying results [38]. NOISeq was chosen because it has been used to accurately detect differentially expressed genes [39].

In the domesticated *Dactylopius coccus*—but also in the wild cochineal *D. opuntiae*—there are different bacteria. Some of them, such as *Wolbachia* and *Spiroplasma*, are commonly found in other insects, while *Dactylopiibacterium* has only been found in the carmine cochineals and has been consistently found there. We expected that *Dactylopiibacterium* would exhibit a differential gene expression in the gut, in comparison to hemolymph or ovaries, considering its large genome (3.6 Mb, containing many transcription regulator genes) and the different conditions that exist in the gut compared to the hemolymph, with different nutrients, pH, and oxygen levels.

LysR transcriptional regulators have an important role in rhizobial symbiosis by turning on nodulation genes [40,41]. In the betaproteobacterium *Burkholderia cenocepacia*, a LysR-type regulator controls morphology and virulence [42]. LysR transcriptional regulators may also regulate genes with diverse functions related to metabolism, motility, and amino acid transport, among others [43]. In *Dactylopiibacterium* there are 29 LysR regulators, and few of them are expressed in both the gut and the hemolymph. *Dactylopiibacterium* has many other transcription regulators that could be involved in a differential expression, leading to only a fraction of its genome being expressed under particular conditions.

### 4.1. Genes Expressed in the Hemolymph and Gut

Genes encoding most ribosomal proteins, tRNA ligases, as well as for the biosynthesis of all the amino acids (except asparagine), were found expressed in the gut and hemolymph, as well as unspecific amino acid transporters, with efflux or influx depending on the gradient between the bacteria and the medium, as occurs with other transporters [44].

In *Dactylopiibacterium*, the large expression of chaperonin genes, also observed in other insect endosymbionts [45,46,47,48] and bacteria with small genomes [49], could be indicative of the close interaction between *Dactylopiibacterium* and its host. GroEL, besides being a chaperonin, has versatile roles in the bacteria–insect interactions [50].

We found different hypothetical genes expressed in both the gut and the hemolymph. By being sap-feeders, sucking insects may ingest a diversity of plant-derived molecules, which are particularly diverse. In plant-associated bacteria, many hypothetical genes expressed in plant roots may participate in transporting and degrading many unknown substances from plants [51]. Proline is an amino acid from plant exudates and has been detected in the phloem of the prickly pear cactus *O. ficus-indica* obtained with *Dactylopius opuntiae* stylets, although in low quantities compared to other amino acids [10]. The *putA* encoding proline dehydrogenase required for proline metabolism is normally induced by proline; we suppose that proline is a *Dactylopiibacterium* nutrient. We found the *putA* gene expressed in *Dactylopiibacterium* in both the hemolymph and in the gut.

Concerning nitrogen fixation regulation, sigma 54 transcriptional regulators that are needed by polymerase to transcribe *nif g*enes were found to be expressed in the hemolymph, gut, and ovaries, supporting that nitrogen fixation may occur in all three tissues. Indeed, N fixation was previously reported from the hemolymph and the ovary [17]. The hydrogen necessary for N fixation would be high in the gut, since it is derived from metabolic processes therein, and the low oxygen found in arthropod guts [52] is favorable for nitrogen fixation. The *nif* gene regulator P II, expressed during nitrogen fixation in the related *Azoarcus* [53], was found expressed in the gut and hemolymph; the *fixA* gene encoding the electron donor for nitrogenase, and molybdenum, iron, and sulfate transporter genes were expressed, as well in the gut and hemolymph. Molybdenum and iron are required for nitrogenase activity [54]. Additionally, the transcripts for cbb3-cytochrome c oxidase were found in the hemolymph. This enzyme is known to be up-regulated in proteobacteria when fixing nitrogen in symbiosis in an anaerobic metabolism [55]. A gene encoding fumarate reductase, which is key in reducing quinones in anaerobiosis, was found to be expressed. Glutamine synthetase participates in ammonium assimilation, and its gene was found expressed in the gut and hemolymph.

### 4.2. Up-Regulated Genes in the Gut

The carmine cochineal feeds on phloem sap, which provides the nutrients that would be found in the insect gut. Sap-sucking insects have been used to analyze the composition of plant phloem [56]. The most abundant amino acids in the phloem of *O. ficus-indica*, obtained with *D. opuntiae* stylets, were valine, isoleucine, leucine, glycine, and tyrosine. On the other hand, threonine, cysteine, and histidine were not detected [10]. By producing proteases and peptidases in the gut, *Dactylopiibacterium* would release additional amino acids from phloem peptides to the host. *Dactylopiibacterium*-expressed genes for proteases and peptidases were found in the gut.

In the gut, there may be other various competing gut bacteria that would not be found in hemolymph, and this may explain the gene expression that leads to the production of the antibiotic colicin (Table S3c). Bacteria normally compete for iron, and this seems to occur in the gut, as iron acquisition genes were found to be highly expressed. There, hemin uptake genes were found up-regulated. Also found in the genome, though not highly expressed, was a hemin degrading factor gene. Isochorismate synthase, whose gene is highly expressed in *Dactylopiibacterium* in the gut, may be used to produce a siderophore as in *Pseudomonas* [57]. This is relevant, as animals keep Fe levels low by conjugating iron, which becomes unavailable to bacteria [58].

From our previous metagenome data from the carmine cochineal [17], we remarked that there were genes for tolerance to metals, both in *Dactylopiibacterium* and in *Wolbachia*. Here, we found that some of these were expressed even in the ovaries, suggesting that the insect ingests metals from the cactus. Numerous plants, such as *O. ficus-indica*, are known for accumulating metals, and have been used for bioremediation [59,60].

Expressed genes for polysaccharide degradation (rhamnogalacturonan I and hemicelluloses) were identified in *Dactylopiibacterium* from the insect gut and hemolymph. Pectins are complex polysaccharides formed by homogalacturonan (α-1,4-linked d-galacturonic acid) polymer backbones with different decorations [61]. Rhamnogalacturonan I is a complex polysaccharide from plant cell walls and mucilage, which contains a linear backbone composed of diglycosyl repeating unit -4)-α-d-Galactopyranose-(1-2)-α-l-Rhamnopyranose [62,63], and has been identified in the prickly pear cactus *O. ficus-indica* [64]. Degradation of rhamnogalacturonan relies on multiple extracellular PL and GH enzymes [65]. In other cases, in honeybees, pectin degradation from the pollen occurs in the midgut by well-adapted γ-proteobacteria by pectate lyases [66]. Expression of both exo- and endo-pectinases, as well as rhamnogalacturonases, was detected in dactylopiibacteria from the gut and hemolymph, suggesting that they are capable of degrading this plant polymer. Additionally, enzymes for hemicellulose degradation (GH74) were detected in *Dactylopiibacterium* in the gut. As described for *Erwinia crysanthemi*, the expression of genes needed for degrading polymers, such as pectins or cellulose, can be induced by the polymer itself or the monomeric sugar units that constitute them (i.e., glucose [67]); this could explain why we have found polymer degradation genes in hemolymph. The related endophytic β-proteobacterium *Azoarcus* sp. BH72 has β-glucosidases and cellobiohydrolases for cellulose degradation [68]. Since there are diurnal fluctuations in non-starch polysaccharides that the insect may consume from the plant [10], we expect that gene expression of *Dactylopiibacterium* carbohydrate catabolic genes may change as well.

### 4.3. Differentially Expressed Genes in Hemolymph

While plant polysaccharides are unlikely to be found in hemolymph, their degradation products (for example, galacturonic acids like glucoronate) may be found in the hemolymph and used as carbon sources by *Dactylopiibacterium*. Additionally, C4-organic acids may fuel nitrogen fixation, as occurs in other nitrogen-fixing symbionts, such as rhizobia in nodules in legumes [69,70]. In hemolymph, there is proline [71]. *Wolbachia*, which is found in the carmine cochineal as well, also has *putA* for proline degradation [16]. Maybe *Wolbachia* populations, quite large indeed (data not shown), compete with *Dactylopiibacterium* for proline. However, nitrogen fixation could confer an advantage to *Dactylopiibacterium* to contend with wolbachia competition for nitrogen nutrients. Notably, proline was a nutrient that, when added to the culture medium of a plant-associated bacterium, enhanced excreted riboflavin production [72]. A hypothesis was reported that suggested that riboflavin production was indicative of nitrogen fixation in plant bacteria [73]. Though the environments may seem unrelated, plant bacteria have many commonalities to insect bacteria [74], pertinently nitrogen fixation. It is worth noting that only two key enzymatic functions are needed to be up-regulated in *Bacillus* to overproduce riboflavin [75]. Here, we found two highly expressed riboflavin-producing genes in hemolymph (Table 1, Tables S1, S2, and S3b).

The up-regulation of genes from the pentose pathway (Table S3b) may serve a double role in *Dactylopiibacterium*. On the one hand, the increased flow in the pentose phosphate pathway would promote riboflavin production [75]—on the other hand, it may contribute to tolerate acid conditions that may be found in hemolymph due to carminic acid. Similarly, in a legume symbiont, the pentose pathway has been implicated in acid tolerance [76].

Since ammonium assimilating genes are found transcribed in *Dactylopiibacterium*, it seems that it may excrete amino acids instead of ammonium to the host. Genes for aromatic amino acids transport found expressed in hemolymph bacteria suggest that they are the providers of essential amino acids to the host. A spermidine synthase gene was up-regulated in hemolymph (Tables S2 and S3b), and spermidine biosynthesis and transport genes were found in the genome of *Sodalis*, which is a secondary endosymbiont in the related wax cochineal [77]. Spermidine may contribute to host colonization and has important functions in the bacterium [78]. Furthermore, arginine (that may be synthesized as well from fixed nitrogen) may stimulate spermidine biosynthesis.

We found in *Dactylopiibacterium* the expression of genes encoding Type IV pili (Tfp) and Type 1 and 2 secretion systems (T1SS and T2SS). In *Azoarcus*, Tfps are needed for endophytic host colonization in rice roots [79]. Tfp systems have been identified as mechanisms for adhesion, biofilm formation, twitching motility, and DNA uptake in some bacteria [80]. Experiments with Tfp mutants in the honeybee gut symbiont *Snodgrassella alvi* showed that Tfps are likely used for attachment and biofilm formation on the hindgut epithelium [81]. Transcriptional evidence suggests Tfp are active in *Dactylopiibacterium*, and could be used for symbiotic motility in the hemolymph and ovary, or adhesion to insect tissues or biofilm formation, as in other bacteria [82]. Additionally, *Vibrio cholerae* T2SS can secrete proteins that promote attachment to chitin for colonization of zooplankton, or to mucin for colonizing the intestinal epithelia of mammals [83]. In enteropathogenic *E. coli*, a T2SS secrets a surface lipoprotein for colonization and biofilm formation [84]. This evidence suggests that T2SS could aid *Dactylopiibacterium* in host colonization.

We do not know the exact location of the flagella, but suggest that *Dactylopiibacterium* may have a single polar flagellum, as in *Azoarcus taiwanensis*, which is related to *Dactylopiibacterium* [85]. Bacterial motility in hemolymph is supported by the high expression of genes for flagellar movement (Figure 4), including genes for the filament, basal body, and motor [86]. In hemolymph, insect cell density is in general low, and dactylopiibacteria may move (as proposed in *Salmonella* [87]) to reach targets such as ovarioles. Therein (in ovaries), dactylopiibacteria may lose flagella, but could use twitching motility instead.

## 5. Conclusions

The quite constant ratios of transcript read numbers of genes involved in transcription, translation, and cell division, as well as the ratios of total genes between hemolymph/gut or hemolymph/ovary comparisons lead us to suggest that there were more *Dactylopiibacterium* cells in hemolymph than in the gut or ovary. In other insects, the number of microbial symbionts may change in different tissues and development stages [88,89]. However, differences in transcript numbers between tissues could be explained, as well by a different metabolic activity of bacteria under these distinct conditions. Further studies to try to quantify dactylopiibacteria inside carmine cochineals are needed.

In plants, nitrogen-fixing endophytes have low numbers in most cases [90,91]. Seemingly *Dactylopiibacterium* emerged from plant endophytes, and similarly in the cochineals; dactylopiibacteria are low in numbers, especially so in ovaries. Nevertheless, therein only a few cells would be needed to colonize new cochineal generations. In-agreement fluorescent *in situ* hybridization (FISH) of *Dactylopiibacterium* in the ovaries showed low numbers of these bacteria [17]. The low number of transcripts from ovaries preclude a differential gene expression analysis, but clearly support a mother–offspring transfer of *Dactylopiibacterium*.

Our interpretation of transcriptomic results is that not all genes from a biochemical pathway need to become up-regulated, and the ones that become highly expressed may represent the limiting steps in a process to overproduce some products, as has been described before for the biotechnological production of riboflavin [75].

Transcriptomic results are very useful to generate hypotheses on bacterial physiology, and our study provides clues toward culturing *Dactylopiibacterium* in the laboratory. The differential expression of transcriptional regulators that we report here would drive a differential expression of *Dactylopiibacterium* in the gut and hemolymph. Here, we report novel traits that highlight the beneficial role of *Dactylopiibacterium* in its host and reveal other functions that will be further studied. Altogether, our results support that the primary symbiont in the cochineal is *Dactylopiibacterium*.

## Figures and Tables

**Table 1:** Top differentially expressed genes.

Protein ID	Log2FC	COG	Function
**Up-regulated in Gut**			
**WP_095525391.1**	4.10	NA	Hypothetical protein
**WP_095523710.1**	3.51	COG2081	Aminoacetone oxidase family FAD-binding enzyme
**WP_095525172.1**	3.50	COG0300	Short-chain dehydrogenase
**WP_095525875.**	3.17	COG0583	LysR family transcriptional regulator
**WP_095523466.1**	3.04	COG2382	Hypothetical protein
**WP_095525673.1**	3.01	NA	Hypothetical protein
**WP_095525264.1**	2.98	COG0643	Chemotaxis protein CheA
**WP_095524233.1**	2.80	NA	Hypothetical protein
**WP_095525304.1**	2.76	COG2199	Hypothetical protein
**WP_095523625.1**	2.67	COG3419	Pilus assembly protein
**WP_095524852.1**	2.65	COG3895	Hypothetical protein
**WP_095526008.1**	2.60	COG3549	Exonuclease ABC subunit A
**WP_095523085.1**	2.58	COG0523	Cobalamin biosynthesis protein CobW
**WP_095523064.1**	2.57	COG0845	Multidrug transporter subunit MdtA
**WP_095524897.1**	2.56	COG2230	SAM-dependent methylytransferase
**Up-regulated in Hemolymph**			
**WP_095525565.1**	3.18	NA	Hypothetical protein
**WP_095525317.1**	2.52	COG0120	Ribose 5-phosphate isomerase A
**WP_095523550.1**	2.47	COG1702	Phosphate starvation-inducible protein PhoH
**WP_095525561.1**	2.41	NA	Hypothetical protein
**WP_095523253.1**	2.39	COG0837	Glucokinase
**WP_095525928.1**	2.36	COG0171	NAD+ synthase
**WP_095523935.1**	2.31	COG1354	Segregation/condensation protein A
**WP_095522941.**	2.13	COG0424	Septum formation inhibitor Maf
**WP_095523049.1**	2.11	COG2200	Hypothetical protein
**WP_095525579.1**	2.11	COG0848	Biopolymer transporter ExbD
**WP_095525717.**	2.09	NA	Thioredoxin
**WP_095526004.1**	2.08	NA	Hypothetical protein
**WP_095524367.1**	2.08	COG1391	Bifunctional [glutamate-amonia ligase]-Adenylyl-l-tyrosine phosphorylase/[glutamate-ammonia-ligase] adenylyltransferase
**WP_095523236.1**	2.06	COG2010	Cytochrome C
**WP_095524731.1**	2.05	NA	GNAT family N-acetyltransferase

NA = No assignation recovered.

## Supplementary Materials

The following are available online at http://www.mdpi.com/2075-1729/9/1/4/s1, Figure S1: Heatmap showing expression level of flagellar, pili or chemotaxis genes. Table S1 Expression levels of *Dactylopiibacterium* genes from different insect tissues. Table S2: Genes differentially expressed with NOISeq analysis. Table S3: Quantitative transcript analysis.
