# Supplementary material for: Metatranscriptomic Analysis of the Bacterial Symbiont Dactylopiibacterium carminicum from the Carmine Cochineal Dactylopius coccus (Hemiptera: Coccoidea: Dactylopiidae)
Source: Life (Basel). 2019 Jan 3;9(1):4. doi: 10.3390/life9010004 (PMC6463064; doi:10.3390/life9010004)
Supplement: Supplementary file 1 [file life-09-00004-s001.zip › supplementary_files-revise/Figure_S1.pdf]

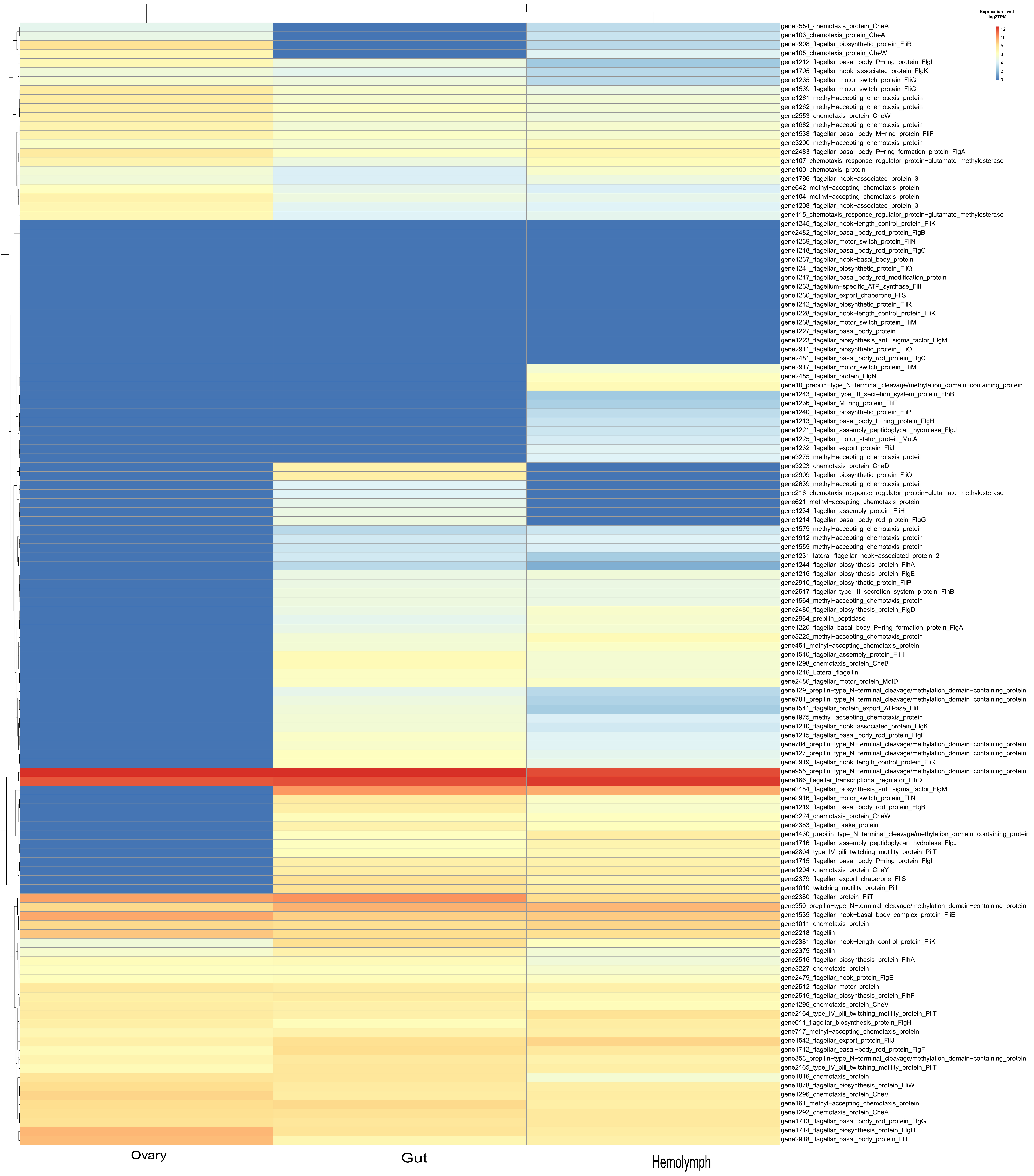

Supplementary Figure 1. Heatmap showing expression level of flagellar, pili or chemotaxis genes.
